# Role of Scar and Border Zone Geometry on the Genesis and Maintenance of Re-Entrant Ventricular Tachycardia in Patients With Previous Myocardial Infarction

**DOI:** 10.3389/fphys.2022.834747

**Published:** 2022-03-24

**Authors:** Vincenzo Gionti, Simone Scacchi, Piero Colli Franzone, Luca F. Pavarino, Roberto Dore, Cesare Storti

**Affiliations:** ^1^Divisione di Cardiologia, Istituto di Cura Città di Pavia, Pavia, Italy; ^2^Dipartimento di Matematica, Università degli Studi di Milano, Milan, Italy; ^3^Dipartimento di Matematica, Università degli Studi di Pavia, Pavia, Italy

**Keywords:** cardiac re-entry, monodomain model, infarct border zone, monomorphic ventricular tachycardia, myocardial infarction

## Abstract

In patients with healed myocardial infarction, the left ventricular ejection fraction is characterized by low sensitivity and specificity in the prediction of future malignant arrhythmias. Thus, there is the need for new parameters in daily practice to perform arrhythmic risk stratification. The aim of this study is to identify some features of proarrhythmic geometric configurations of scars and border zones (BZ), by means of numerical simulations based on left ventricular models derived from post myocardial infarction patients. Two patients with similar clinical characteristics were included in this study. Both patients exhibited left ventricular scars characterized by subendo- and subepicardial BZ and a transmural BZ isthmus. The scar of patient #1 was significantly larger than that of patient #2, whereas the transmural BZ isthmus and the subdendo- and subepicardial BZs of patient #2 were thicker than those of patient #1. Patient #1 was positive at electrophysiologic testing, whereas patient #2 was negative. Based on the cardiac magnetic resonance (CMR) data, we developed a geometric model of the left ventricles of the two patients, taking into account the position, extent, and topological features of scars and BZ. The numerical simulations were based on the anisotropic monodomain model of electrocardiology. In the model of patient #1, sustained ventricular tachycardia (VT) was inducible by an S2 stimulus delivered at any of the six stimulation sites considered, while in the model of patient #2 we were not able to induce sustained VT. In the model of patient #1, making the subendo- and subepicardial BZs as thick as those of patient #2 did not affect the inducibility and maintenance of VT. On the other hand, in the model of patient #2, making the subendo- and subepicardial BZs as thin as those of patient #1 yielded sustained VT. In conclusion, the results show that the numerical simulations have an effective predictive capability in discriminating patients at high arrhythmic risk. The extent of the infarct scar and the presence of transmural BZ isthmuses and thin subendo- and subepicardial BZs promote sustained VT.

## 1. Introduction

Sudden cardiac death (SCD) after myocardial infarction is a significant public healthcare burden (Fishman et al., 2010). The main goal of clinical electrophysiology in the primary prevention of major arrhythmic events is the development of accurate risk stratification algorithms. Based on the results from clinical trials, left ventricular ejection function (LVEF) and patient New York Heart Association (NYHA) functional classification are used as the principal diagnostic parameters in designing treatments to avoid future sudden cardiac death (Priori et al., 2015; Al-Khatib et al., 2018). However, other studies (Gorgels et al., 2003; Stecker et al., 2006; Sabbag et al., 2015) raise skepticism about the true role of LVEF, because this approach is characterized by low sensitivity and specificity in the prediction of malignant arrhythmias. To date, in patients with remote myocardial infarction and preserved LVEF, no non-invasive risk stratification technique has demonstrated sufficient specificity and sensitivity. Although a two-step approach including non-invasive risk factors (such as premature ventricular complexes, non-sustained ventricular tachycardia, late potentials, and prolonged QTc), and electrophysiology study (EPS) can help in the stratification of arrhythmic risk (see Gatzoulis et al., 2019), in the near future, new effective and non-invasive parameters need to be introduced for arrhythmic risk stratification. For this purpose, a full understanding of the role of each arrhythmic substrate component is necessary for outcome prediction. Besides the clinical and experimental studies, computer modeling might represent a reliable and effective tool to predict the arrhythmic risk of specific patients and the ablation target, as proposed in several recent publications (Ashikaga et al., 2013; Deng et al., 2015, 2016; Arevalo et al., 2016; Campos et al., 2021).

The scar tissue and the border zone (BZ) constitute the most common arrhythmogenic substrate in ischemic cardiomyopathy (Moran et al., 1982). In this regard, cardiac magnetic resonance (CMR) is a highly effective imaging modality for the characterization of myocardial tissue (Pattanayak and Bleumke, 2015). Histologically, a myocardial scar is not always a uniform lesion. In fact, the fibrosis areas in the scar are interrupted by viable fibers that constitute slow conduction pathways (Fenoglio et al., 1983). It is well recognized that scar heterogeneity within the myocardium at CMR is arrhythmogenic (Peters et al., 1997; Peters and Wit, 1998; de Bakker et al., 2005; Ciaccio et al., 2007). Furthermore, in most cases, stable ventricular tachycardia (VT) circuits have two scarred areas and a central isthmus or channel composed of a small mass of viable fibers (Codreanu et al., 2008).

Investigating the role of scar and BZ electrophysiological and geometric properties on the onset and maintenance of re-entry dynamics is an active research area; refer to the experimental studies (Wit et al., 1982; Dillon et al., 1988; Janse and Wit, 1989; Peters et al., 1997). Recent computational studies have investigated the pro-arrhythmic mechanisms associated with BZ tissue (Cabo and Boyden, 2003; Ciaccio et al., 2004, 2007, 2015; Cabo et al., 2006; Cabo, 2014; Connolly et al., 2015; Connolly and Bishop, 2016; Campos et al., 2018).

Our previous numerical study (Colli-Franzone et al., 2019) demonstrated, in idealized conditions, that configurations characterized by a thin subepicardial BZ and wide scar, even without transmural BZ isthmuses, facilitate the onset and perpetuation of VT re-entry. However, to our knowledge, a detailed study on patient-derived geometric configurations of scars and BZ that are more likely to promote VT is still missing in the literature.

The aim of the present investigation is to identify some features of proarrhythmic geometric configurations of scar and BZ, by means of numerical simulations based on left ventricle models derived from two post-infarction patients, similar with respect to age, gender, NYHA class, the prevalence of coronary artery disease and scar localization, but different in the outcome of EPS.

## 2. Methods

We applied a four-step model: (1) selection of post-myocardial infarction patients with moderate systolic dysfunction and different vulnerability to VT at EPS; (2) identification at CMR of scar and BZ anatomic pattern of interest; (3) formulation of a mathematical model of the left ventricle, taking into account the position, extent, and topological features of the scars and BZ; and (4) modulation of the geometric features of scars and BZ.

### 2.1. Clinical Data

We retrospectively screened patients with prior myocardial infarction scheduled for an EPS between January 1, 2019, and July 1, 2020. The patients did not meet criteria for ICD implantation in primary prevention. The reason for referral to the EPS was arrhythmia risk stratification because of moderate left ventricular dysfunction at CMR and a history of frequent premature ventricular contractions and syncope. Of the seven patients that were screened, two patients were included because they were similar with respect to age, gender, the prevalence of coronary artery disease, scar localization, but different in EPS outcome.

For patients selection, a comprehensive patient medical history, including coronary artery disease risk factors, NYHA class, and medications at the time of EPS, was obtained. Additionally, 12-lead electrocardiography was performed and interpreted in combination with clinical and CMR data. Myocardial infarction was considered if there were previous symptoms of myocardial ischemia, pathological Q waves at 12-lead electrocardiography, loss of viable myocardium at CMR in a pattern consistent with an ischaemic etiology, and a history of a rise and/or fall of cardiac troponin (cTn) values with at least one value above the 99th percentile upper reference limit (URL), in line with a universal definition of myocardial infarction (Thygesen et al., 2018). The clinical characteristics of the two patients are reported in Table 1.

**Table 1:** Clinical characteristics of the two patients.

**Clinical characteristics**	**EPS positive**	**EPS negative**
NYHA class	II	II
Age	57 years old	59 years old
Gender	Male	Male
Hypertension	Yes	Yes
Dyslipidemia	Yes	Yes
Diabetes Mellitus	Yes	No
Tabagism	Yes	No
Obesity^*^	No	No
Time from MI to EPS	12 months	27 months
No. of coronaropathy	2 vessels	2 vessels

* *Obesity was defined as BMI ≥ 30 Kg/m^2^*.

#### 2.1.1. CMR Acquisition

Clinical 3-T scanners (Siemens Sonata) with phased-array receiver coils and standard protocols were used. Briefly, cine images were acquired in multiple short-axis (every 10 mm throughout the entire LV) and 3 long-axis views using a steady-state free precession sequence (slice thickness, 6 mm; inter-slice gap, 4 mm; TR, 3.0 ms; TE, 1.5 ms; temporal resolution, 35–40 ms; flip angle, 60; in-plane resolution 1.71.4 mm). Delayed enhancement cardiovascular magnetic resonance (DE-CMR) was performed using a segmented inversion-recovery gradient-echo sequence (slice thickness, 6 mm; inter-slice gap, 4 mm; TR, 9.5 ms; TE, 3.8 ms; flip angle, 25; in-plane resolution 1.8x1.4 mm) 10 min after contrast administration (gadoversetamide, 0.15 mmol/kg) in the identical locations as cine-CMR. The inversion delay time was set to null signal from normal myocardium and was typically 280–360 ms.

#### 2.1.2. CMR Analysis

**Figure 1:**
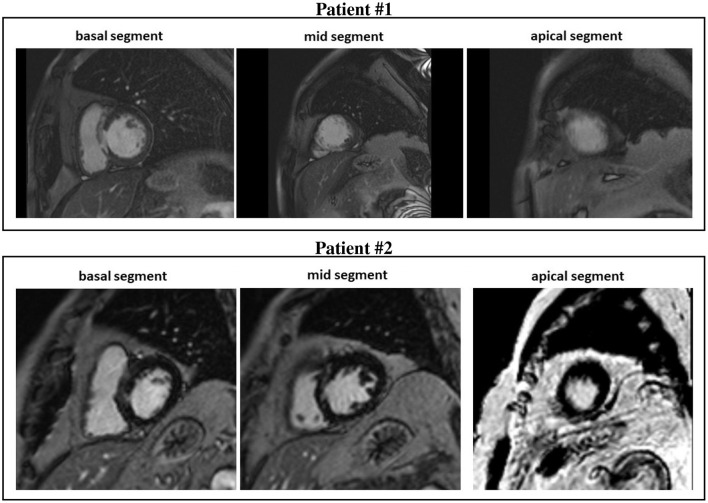
Cardiovascular magnetic resonance (CMR) short-axis slices of the basal, mid, and apical segment of patients #1 and #2.

**Figure 2:**
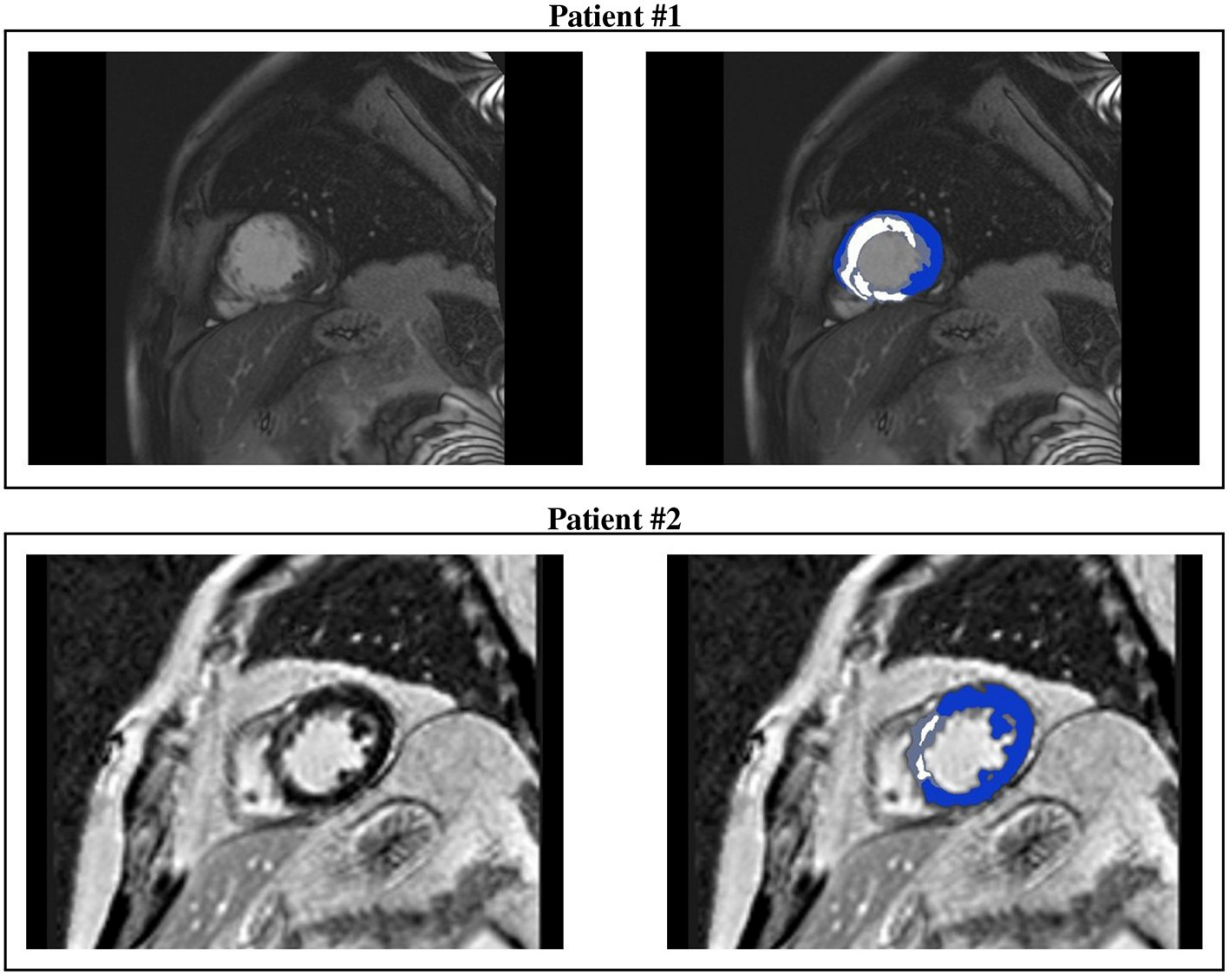
CMR short-axis slices (left) of interest of patients #1 and #2 and the resulting ventricular segmentation (right) into non-infarcted myocardium (blue), border zone (gray), and scar (white).

All CMR analyses were performed with Digital Imaging and Communications in Medicine (DICOM) images with a custom software package. Left ventricular volumes, mass, and ejection fraction were quantitatively measured from the stack of short-axis cine images using standard techniques (Rehr et al., 1985; Walsh and Hundley, 2007). Papillary muscles were regarded as part of the ventricular cavity. For each patient, the maximum signal intensity (SI) within an infarct region in each image of the left ventricle stack was automatically determined, and the scar was defined as myocardium with SI > 50% of the maximal SI. A region of interest was then placed by a trained observer in the remote myocardium, i.e., a portion of myocardium without hyperenhancement and with normal motion, in an area free of artifacts and with uniform myocardial suppression. The signal suppression allows the objective spatial extension of the hyperenhancement area. BZ within the infarct periphery was defined as the myocardium with SI > peak remote SI but SI <50% of maximal SI of the high SI myocardium (Amado et al., 2004; Schmidt et al., 2007). The transmural extent of hyperenhancement was measured by standard techniques (Kim et al., 2000). Each short-axis slice was segmented circumferentially into 12 wedges. For each segment, the transmural extent of total hyperenhancement was expressed as a percentage of the total segment area. For each patient, the percentage of segments with transmural extents of hyperenhancement within each quartile (0 to 25%; 26 to 50%; 51 to 75%; or > 75%) was determined. Table 2 summarizes the CMR characteristics of the two patients, see Figures 1, 2.

**Table 2:** Cardiovascular magnetic resonance (CMR) features of the two patients.

**CMR characteristics**	**EPS positive**	**EPS negative**
Basal-apex length	94 mm	93 mm
LV end-diastolic volume	228 ml	232 ml
LV end-diastolic diameter	57.26 mm	58.37
Wall thickness	9.2 mm	10.0 mm
LV ejection fraction	37 %	43 %
Isthmus section diameter	1.5 mm	3.1 mm
Scar location	Antero-septal	Antero-septal
Endocardial scar and BZ area	1416.11 mm^2^	824.42 mm^2^
Endocardial BZ thickness	1.2 mm	1.8 mm
Epicardial BZ thickness	1.3 mm	3.1 mm

#### 2.1.3. Electrophysiologic Testing

Electrophysiology study was performed using standard techniques. Briefly, programmed stimulation was performed using two drive trains (600 and 400 ms) followed by one to three ventricular extrastimuli. The extrastimuli were 2 ms in duration at two times the diastolic threshold at two right ventricular sites (apex and outflow tract), reducing the coupling interval until reaching the ventricular refractoriness or a coupling interval of 190 ms. The EPS results were classified as negative (non-inducibility with complete protocol) or positive (monomorphic VT that lasted 30 s or required cardioversion for hemodynamic compromise). Additionally, two expert operators analyzed the superficial ECG morphology of VT and interpreted it in combination with the isthmus localization at CMR. Two patients were included in the study, one with negative EPS and one with positive EPS. Tables 1, 2 summarize the clinical and CMR characteristics, respectively. In both patients, the maximum scar extension is localized in the mid-interventricular septum. The scar extension was greater in the patient with positive EPS, instead, the isthmus thickness was greater in the patient with negative EPS.

### 2.2. Computer Modeling

To model the electrical current flow through the myocardium, we assume the monodomain representation of the cardiac tissue (Colli-Franzone et al., 2014). The computational electrophysiological model considers three different types of cardiac tissue regions: healthy myocardium, necrotic scar, and the infarct BZ.

Let Ω denotes a three-dimensional portion of the myocardium. According to the monodomain model, the evolution of the transmembrane potential *v*(**x**, *t*), gating variables **w**(**x**, *t*), and ionic concentrations **c**(**x**, *t*) is described by the following system of non-linear partial differential equations:


(1) $$\{\begin{array}{ll} c_{m}\partial_{t}v-div(D\nabla v)+i_{ion}(v,\text{w},\text{c})=i_{app} & \text{ in }\Omega \\ \partial_{t}w-R_{w}(v,\text{w},\text{c})=0,\;\partial_{t}c-R_{c}(v,\text{w},\text{c})=0 & \text{ in }\Omega \\ n^{T}D\nabla v=0 & \text{ on }\partial \Omega \end{array}$$

with appropriate initial conditions on *v*(**x**, 0), **w**(**x**, 0), and **c**(**x**, 0). Here, *c*_*m*_ and *i*_*ion*_ denote the capacitance and the ionic current of the membrane per unit volume, *i*_*app*_ represents the applied current per unit volume, and *D* is the anisotropic conductivity tensors.

Assuming transversely isotropic properties of the intra- and extracellular media, the intra- and extracellular conductivity tensors are given by


(2) $$D_{i,e}(\text{x})={\text{σ}}_{\text{t}}^{\text{i},\text{e}}\;\text{I}+({\text{σ}}_{\text{l}}^{\text{i},\text{e}}-{\text{σ}}_{\text{t}}^{\text{i},\text{e}}){\text{a}}_{\text{l}}(\text{x})\;{\text{a}}_{\text{l}}{\left(\text{x}\right)}^{\text{T}},$$

where $\sigma_{l}^{i,e}$ and $\;\sigma_{t}^{i,e}$ are the conductivity coefficients of the intra- and extracellular media measured along the fiber direction **a**_*l*_(*x*) and any cross fiber direction, respectively. According to the Monodomain model derivation presented in Colli-Franzone et al. (2005), the tensor *D* is given by


$$\text{D}(\text{x})={\text{D}}_{\text{e}}(\text{x}){\left({\text{D}}_{\text{i}}(\text{x})+{\text{D}}_{\text{e}}(\text{x})\right)}^{-1}{\text{D}}_{\text{i}}(\text{x})={\text{σ}}_{\text{t}}\text{I}+({\text{σ}}_{\text{l}}-{\text{σ}}_{\text{t}})\;{\text{a}}_{1}(\text{x}){\text{a}}_{1}{\left(\text{x}\right)}^{\text{T}}$$

where $\sigma_{t,l}=\frac{\sigma_{t,l}^{e}\sigma_{t,l}^{i}}{\sigma_{t,l}^{e}+\sigma_{t,l}^{i}}$.

The ionic current is given by *i*_*ion*_ = χ*I*_*ion*_, where χ is the membrane surface to volume ratio and the ionic current per unit area of the membrane surface *I*_*ion*_ is given by the ten Tusscher membrane model (TP06) (ten Tusscher et al., 2004; ten Tusscher and Panfilov, 2006), available from the cellML depository (models.cellml.org/cellml). The TP06 ionic model also specifies the functions *R*_*w*_(*v*, **w**) and *R*_*c*_(*v*, **w**, **c**) in the ordinary differential equations (ODEs) system, consisting of 17 ODEs modeling the main ionic currents dynamics.

### 2.3. Numerical Methods

The space discretization of the system (1) is performed by employing hexahedral isoparametric *Q*_1_ finite elements, while the time discretization is based on splitting the ODEs of the membrane model from the reaction-diffusion PDE. Regarding the PDE, a semi-implicit scheme is adopted, where the diffusion term is treated implicitly, while the reaction term is treated explicitly. This discretization strategy yields a large-scale linear system of algebraic equations that must be solved at each time step. In order to ensure parallelization and portability of our Fortran code, we use the PETSc parallel library (Balay et al., 2020), a suite of data structures and functions for building large-scale parallel scientific applications, based on the MPI communication library. The parallel strategy employed is based on a geometric domain decomposition strategy, where each subdomain is assigned to one processor and the information associated with the interior of the subdomain is uniquely owned by that processor. The processor stores all subvectors and a block of the matrices (mass, stiffness) associated with each subdomain. The linear system at each time step is solved by a parallel conjugate gradient method, preconditioned by the Block Jacobi preconditioner, with ILU(0) local solvers. For further details about the numerical procedure, refer to our previous works (Colli-Franzone et al., 2011, 2014, 2019). The simulations are run on 128 cores of the Linux Cluster Galileo of Cineca.

### 2.4. Simulations Setup

**Figure 3:**
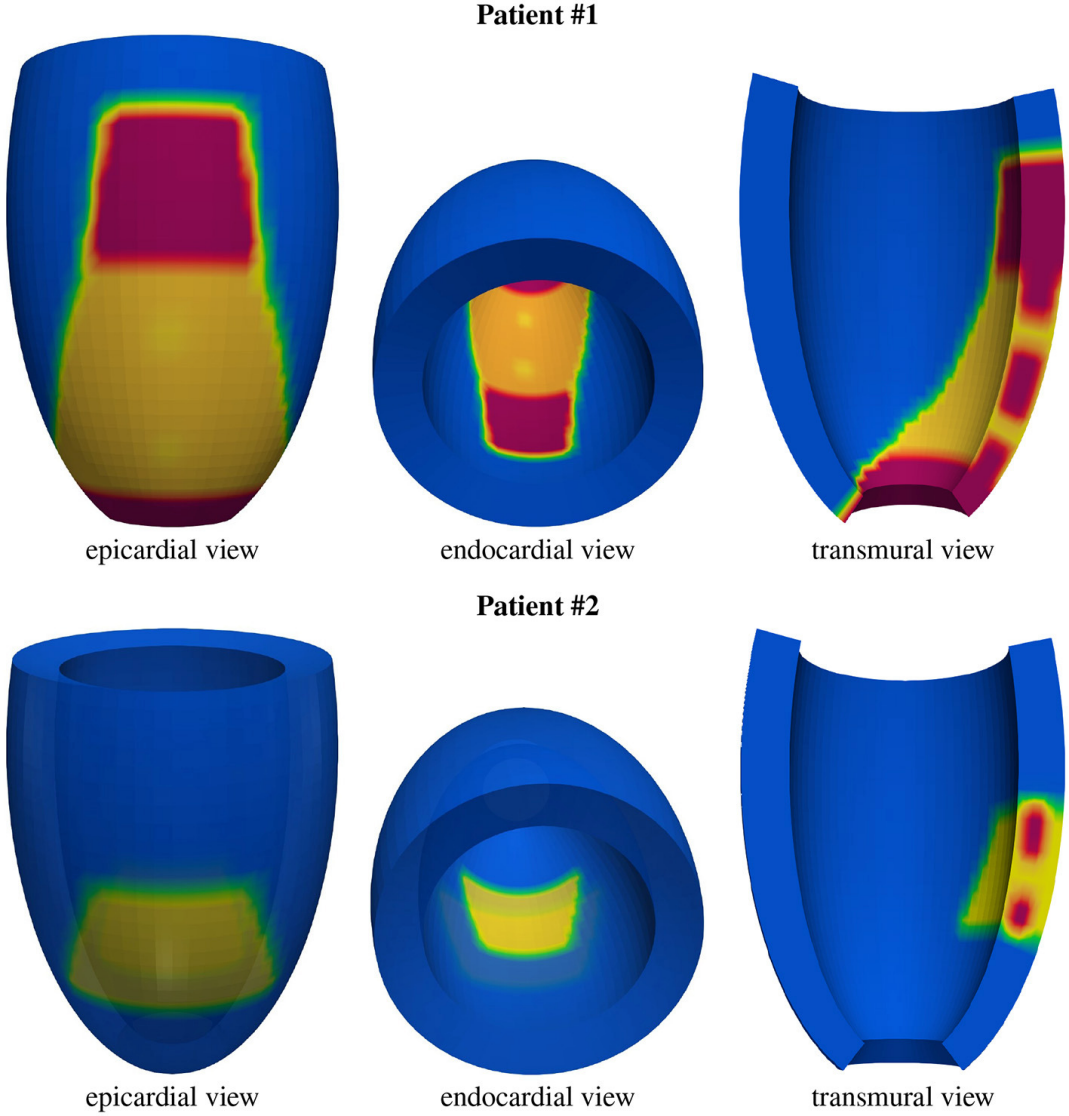
Geometric models of the left ventricles of the two patients: healthy tissue (blue), border zone (BZ) tissue (yellow), scar tissue (red). From the transmural view, one can appreciate the transmural BZ isthmuses of the two patients.

**Computational domain**. The domain *H* is the image of a Cartesian periodic slab using ellipsoidal coordinates, yielding a truncated ellipsoid modeling a left ventricle (LV) geometry, described by the parametric equations


$$\{\begin{array}{ll} x=a(r)\cos\theta \cos\phi \; & \phi_{min}\leq \phi \leq \phi_{max},\\ y=b(r)\cos\theta \sin\phi \; & \theta_{min}\leq \theta \leq \theta_{max},\\ z=c(r)\sin\theta \; & 0\leq r\leq 1, \end{array}$$

where *a*(*r*) = *a*_1_+*r*(*a*_2_−*a*_1_), *b*(*r*) = *b*_1_+*r*(*b*_2_−*b*_1_), *c*(*r*) = *c*_1_+*r*(*c*_2_−*c*_1_). The parameters *a*_1_ = *b*_1_, *a*_2_ = *b*_2_, *c*_1_, and *c*_2_ are tuned to match the dimension of the left ventricle of the two patients (refer to Figure 3 and Table 3 for the geometric models and the position of scars and BZ), whereas ϕ_*min*_ = −π/2, ϕ_*max*_ = 3π/2, θ_*min*_ = −3π/8, and θ_*max*_ = π/8. We will refer to the inner surface of the truncated ellipsoid (*r* = 0) as endocardium and to the outer surface (*r* = 1) as epicardium. In all computations, a structured grid of 512 × 256 × 48 hexahedral isoparametric *Q*_1_ finite elements of size *h*≈0.02 *cm* is used in space, for a total amount of 6,447,616 mesh nodes. Fibers rotate transmurally, linearly with the depth and counterclockwise from epicardium to endocardium, for a total amount of 120°. More precisely, in a local ellipsoidal reference system (**e**_ϕ_, **e**_θ_, **e**_*r*_), the fiber direction **a**_*l*_(**x**) at a point **x** is given by


$$\begin{array}{l} {\text{a}}_{l}(\text{x})={\text{e}}_{\phi }\text{cos}\alpha (r)+{\text{e}}_{\theta }\text{sin}\alpha (r),\text{with }\alpha (r)=\frac{2}{3}\pi (1-r)-\frac{\pi }{4},\\ \;0\leq r\leq 1. \end{array}$$

The volume occupied by the left ventricular myocardial tissue of patient #1 is 141.3 *cm*^3^, where 25.3 and 4.3 *cm*^3^ are occupied by the scar and BZ, respectively. The volume occupied by the left ventricular myocardial tissue of patient #2 is 160.1 *cm*^3^, where 6.9 and 5.2 *cm*^3^ are occupied by the scar and BZ, respectively.

**Table 3:** Parameters of the truncated ellipsoidal geometries expressed in *cm*.

**Patient**	***a*** **_1_**	***a*** **_2_**	***b*** **_1_**	***b*** **_2_**	***c*** **_1_**	***c*** **_2_**
#1	2.5	3.5	2.5	3.5	6.5	7.1
#2	2.5	3.6	2.5	3.6	6.5	7.2

**Parameter calibration**. The values of the transversely isotropic conductivity coefficients in (2), that we use for the healthy tissue in all the numerical tests, are $\sigma_{l}^{i}=3$, $\sigma_{t}^{i}=0.31525$, $\sigma_{l}^{e}=2$, and $\sigma_{t}^{e}=1.3514$, all expressed in *mΩ*^−1^*cm*^−1^; refer to Colli-Franzone et al. (1990, 2011) for the derivation of these conservative values. These values, coupled with the TP06 membrane model, predict conduction velocities of about 0.061 and 0.027 cm/ms for excitation layer propagating along and across the fiber direction, respectively. We remark that these conduction velocities are within the physiological range, refer to Roberts et al. (1979) and Punske et al. (2003). The membrane surface to volume ratio is χ = 10^3^ *cm*^−1^ and the membrane capacitance per unit volume is *c*_*m*_ = χ*C*_*m*_, where $C_{m}=1\;\mu F/cm^{2}$ is the membrane capacitance per unit area.

Structural and functional remodeling in healed infarct generates a layer of viable myocardium cells, the so-called BZ (Pinto, 1999; Nattel et al., 2007). The BZ tissue is characterized by scar patches (de Jong et al., 2011; McDowell et al., 2011) and marked electrical and topological heterogeneity (Peters et al., 1997; Ciaccio et al., 2007).

We model the BZ by implementing a 60% reduction of fast sodium current conductance *g*_*Na*_ (Baba et al., 2005; Decker and Rudy, 2010), a 70% reduction of L-type calcium current conductance *g*_*CaL*_ (Baba et al., 2005) and a reduction of 70 and 80% of the potassium currents conductances *g*_*Kr*_ and *g*_*Ks*_, respectively, (Jiang et al., 2000). Previous experimental studies (Yao et al., 2003) have also shown a loss of the average number of transverse gap-junctions between viable fibers in BZ tissue. We model this loss as a 75% reduction of cross fiber intracellular conductivity coefficient $\sigma_{t}^{i}$. Regarding the modeling of BZ tissue properties, we also refer to the review article (Mendonca Costa et al., 2018).

In the scar region, we assume an isotropic conductivity amounting to 0.5*mΩ*^−1^*cm*^−1^. To our knowledge, previous works have considered values of scar conductivity ranging between 0.05 and 0.5 *mΩ*^−1^*cm*^−1^. We have chosen the value of 0.5 because it is the value closest to the average of the conductivities assigned in the BZ.

**Figure 4:**
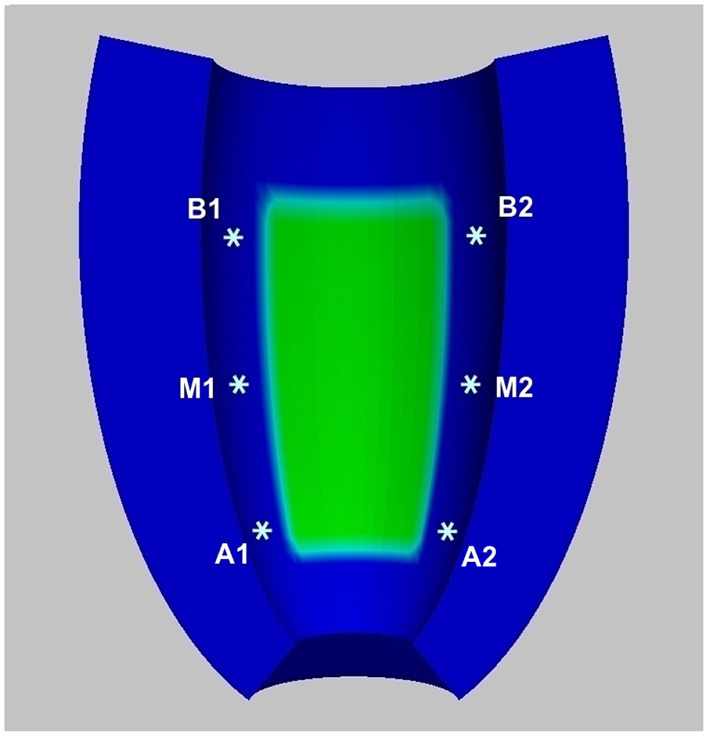
Schematic representation of stimulation sites.

**Stimulation protocol**. Stimulations of 250*mA*/*cm*^3^ amplitude and 1*ms* duration are applied in a small subendocardial volume located in six different sites depending on the simulation points displayed in Figure 4: apical stimulation at points A1 or A2; central stimulation at points M1 or M2; basal stimulation at points B1 or B2. For each stimulation point, we first apply five pacing stimuli (S1) at a basic cycle length (BCL) of 500 *ms*. Then, a premature stimulus (S2) is delivered 350 *ms* after S1. If S2 does not generate a re-entrant arrhythmia, the S1-S2 coupling interval is shortened in steps of 10 *ms* until arrhythmia is induced or the S2 fails to trigger excitation. If an arrhythmia is not induced, an additional S3, and if necessary S4, is delivered in the same manner as S2 (initially delivered 350 *ms* after the previous stimulus, and then shortened until arrhythmia is induced or the stimulus fails). The final simulation time is 4 *s*. We consider a re-entry sustained if it maintains until 4 *s*.

**Postprocessing**. The transmembrane potential distributions reported in the following are generated using Paraview-5.7.0-RC1, whereas the activation time distributions and potential waveforms are generated using Matlab_R2021a. The activation time is defined in the general point **x** as the unique instant *t*_*A*_ when the transmembrane potential *v*(**x**, *t*_*A*_) = −50 *mV* during the depolarization phase of the action potential.

## 3. Results

### 3.1. Patient #1 LV Model

**Figure 5:**
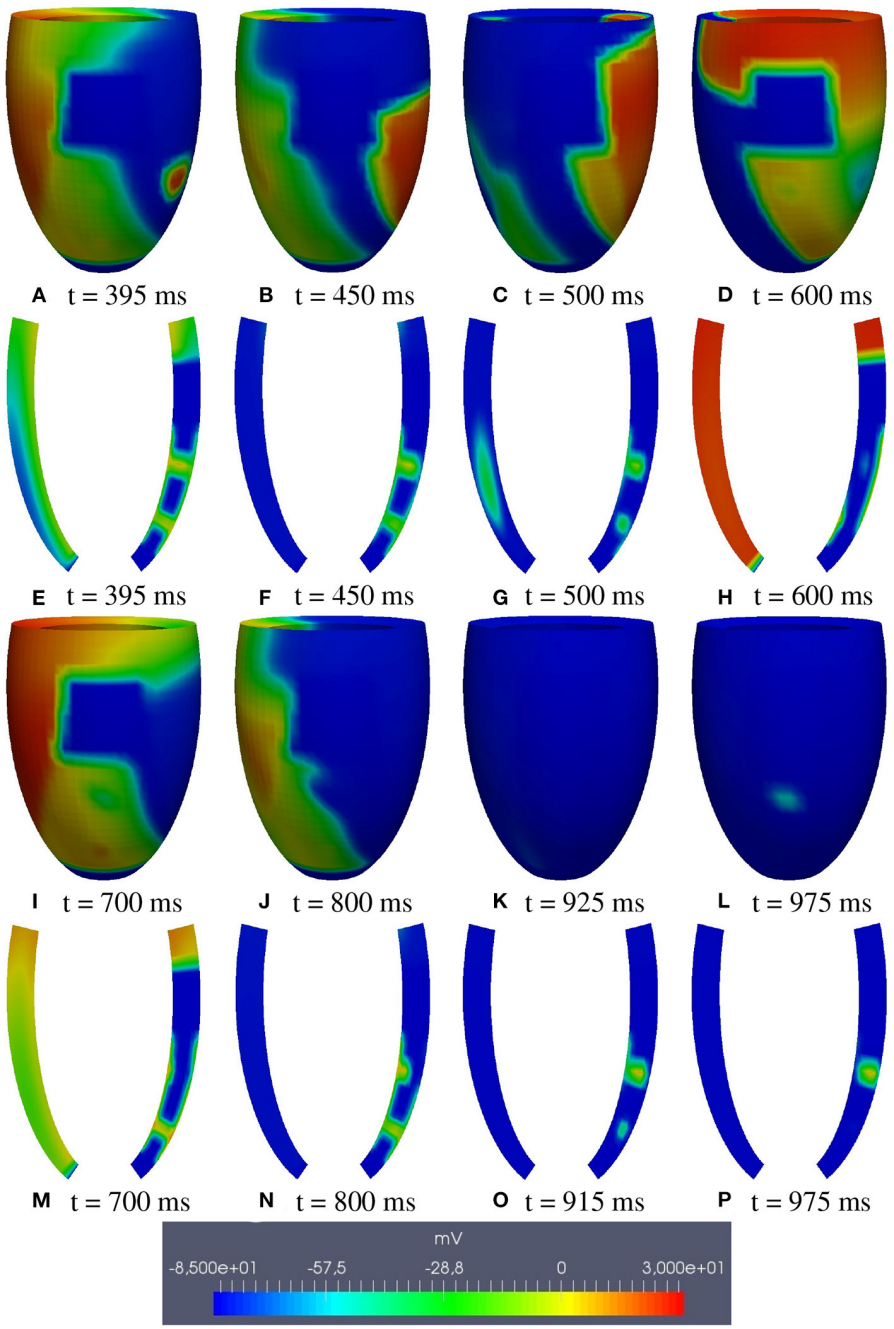
Patient #1. M1 stimulation. **(A–P)** Transmembrane potential snapshots (t = 395–975 ms) on the epicardial surface and on a transmural section. t = 0 corresponds to the S1 stimulus. The S2 stimulus is applied at t = 350 ms.

**Figure 6:**
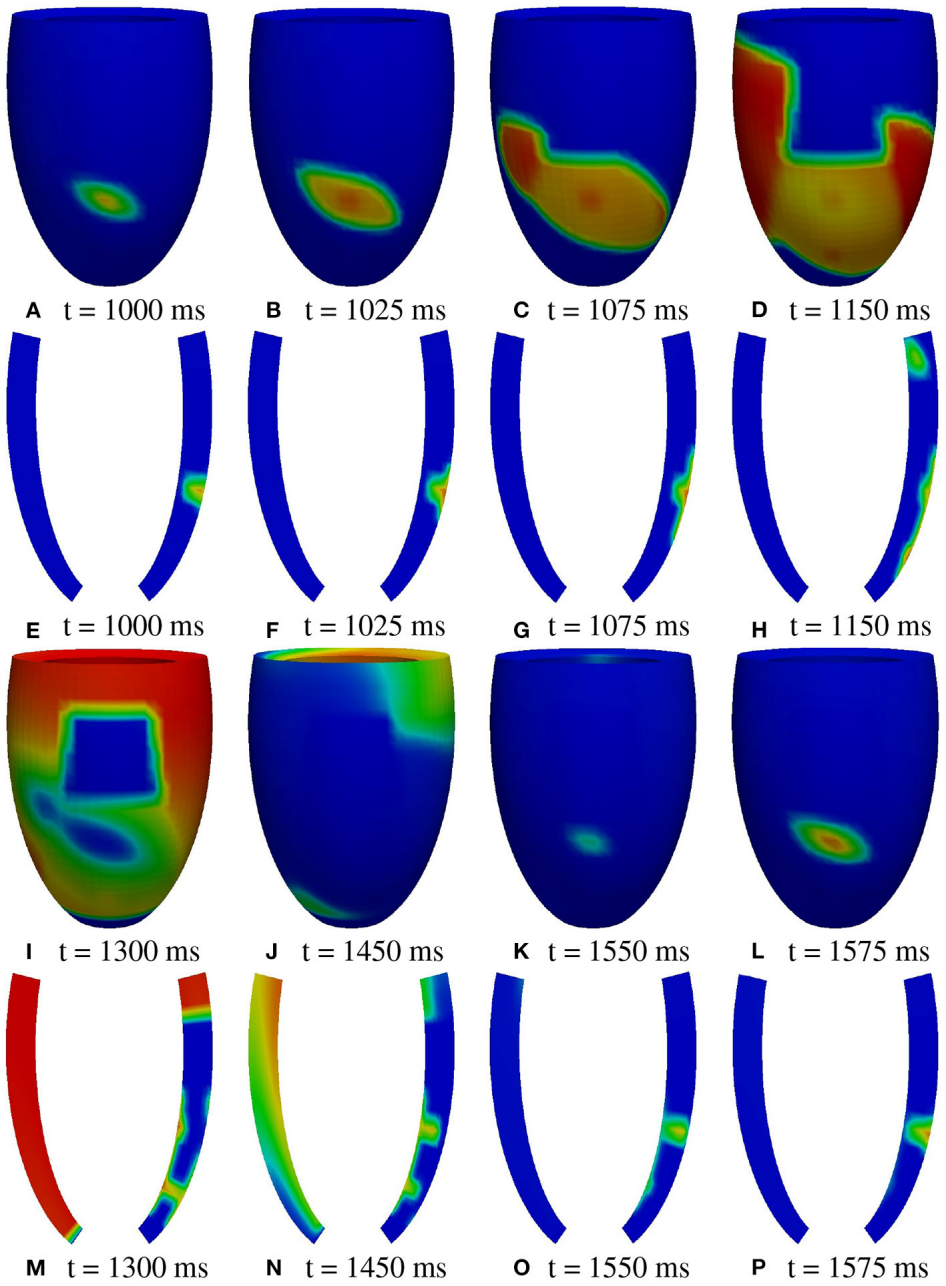
Patient #1. M1 stimulation. **(A–P)** Transmembrane potential snapshots (t = 1,000–1,575 ms) on the epicardial surface and on a transmural section. t = 0 corresponds to the S1 stimulus. The S2 stimulus is applied at t = 350 ms. The colorbar is the same as in Figure 5.

**M1 stimulation site**. Figures 5, 6 report some snapshots of the transmembrane potential on the epicardium and on two longitudinal transmural sections, after the S2 stimulus, applied at t = 350 ms, being t = 0 ms the onset of the S1 stimulus. At t = 395 ms (Figure 5A), the excitation layer elicited by the S2 stimulus reaches the epicardial surface, generating a breakthrough (BKT) on the right side of the scar, halfway between the apex and the base. Then, excitation spreads through epicardium, entering the BZ at t = 450 ms (Figure 5B) and moving around the basal portion of the scar (Figures 5C,D). At t = 600 ms (Figure 5H), both endocardial and epicardial layers enter the apical isthmus, which results in complete activation at t = 700 ms (Figure 5M). At the same time, the endocardial excitation layer starts to enter the central isthmus, while the epicardial excitation layer is blocked at the epicardial side of the central isthmus. The excitation layer propagates very slowly through the isthmus, from the endocardium to the epicardium (Figures 5N,O). At t = 975 ms, excitation exits the BZ subepicardial layer over the central isthmus, generating an epicardial BKT (Figures 5L,P), which induces a propagating quasi-elliptical epicardial excitation layer, with the major axis aligned with the epicardial fiber direction. This excitation layer triggers the first cycle of re-entry (Figures 6A–H). Furthermore, when the excitation layer reaches the healthy myocardial tissue, the propagation proceeds intramurally toward the endocardial surface, moving around the basal scar region. The re-entrant activation follows the same circuit through the central isthmus (Figures 6I–P) for two cycles and then dies. We report in **Figure 9A** the epicardial activation time distributions of the S2 and first reentrant excitation sequence. In order to clarify the dynamics of the excitation sequence at the level of the epicardial surface and transmurally, we have added the movies **SM_paz1_M1_epi** and **SM_paz1_M1_trans** in the Supplementary Material.

**Figure 7:**
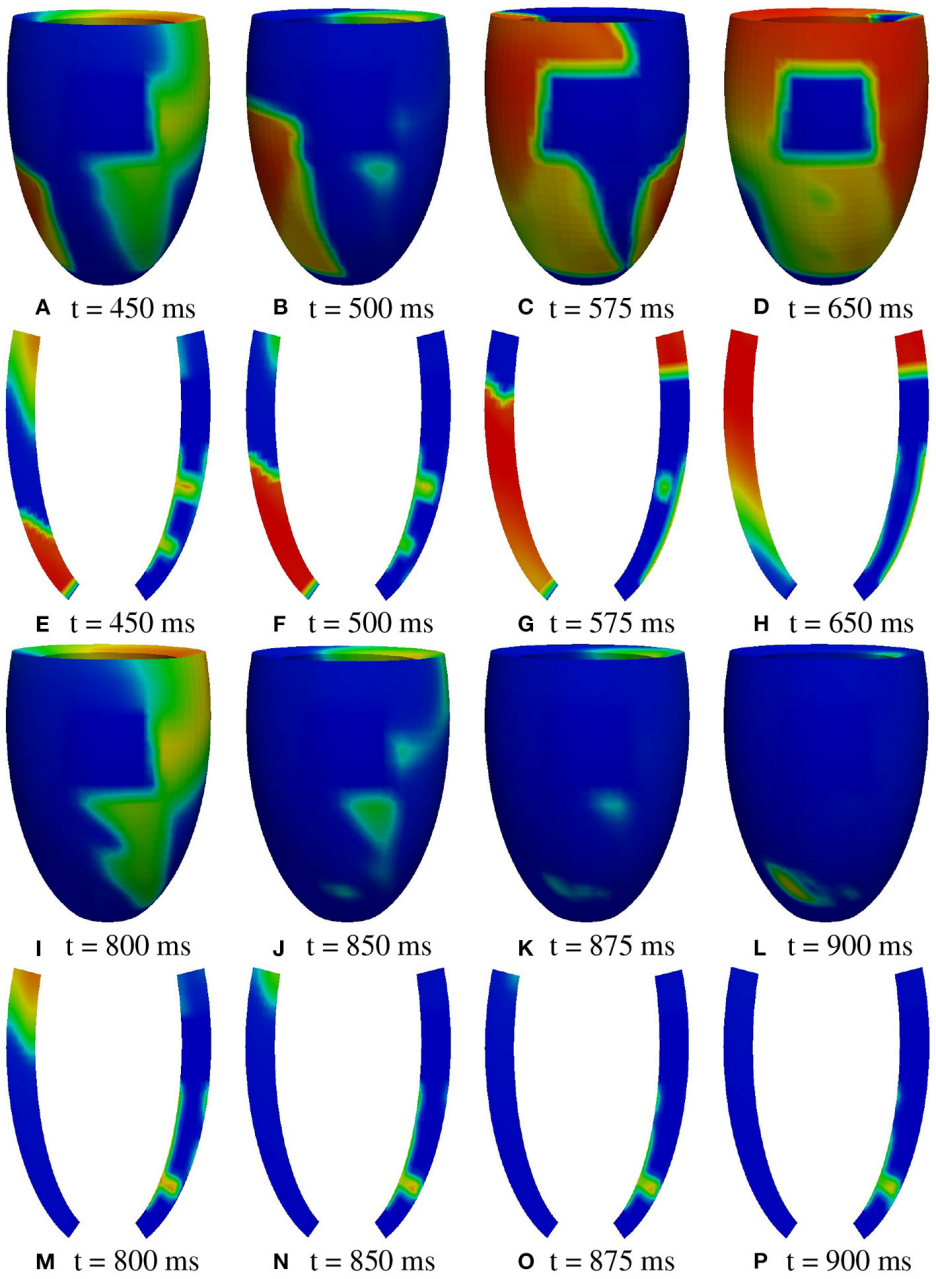
Patient #1. A2 stimulation. **(A–P)** Transmembrane potential snapshots (t = 450–900 ms) on the epicardial surface and on a transmural section. t = 0 corresponds to the S1 stimulus. The S2 stimulus is applied at t = 350 ms. The colorbar is the same as in Figure 5.

**Figure 8:**
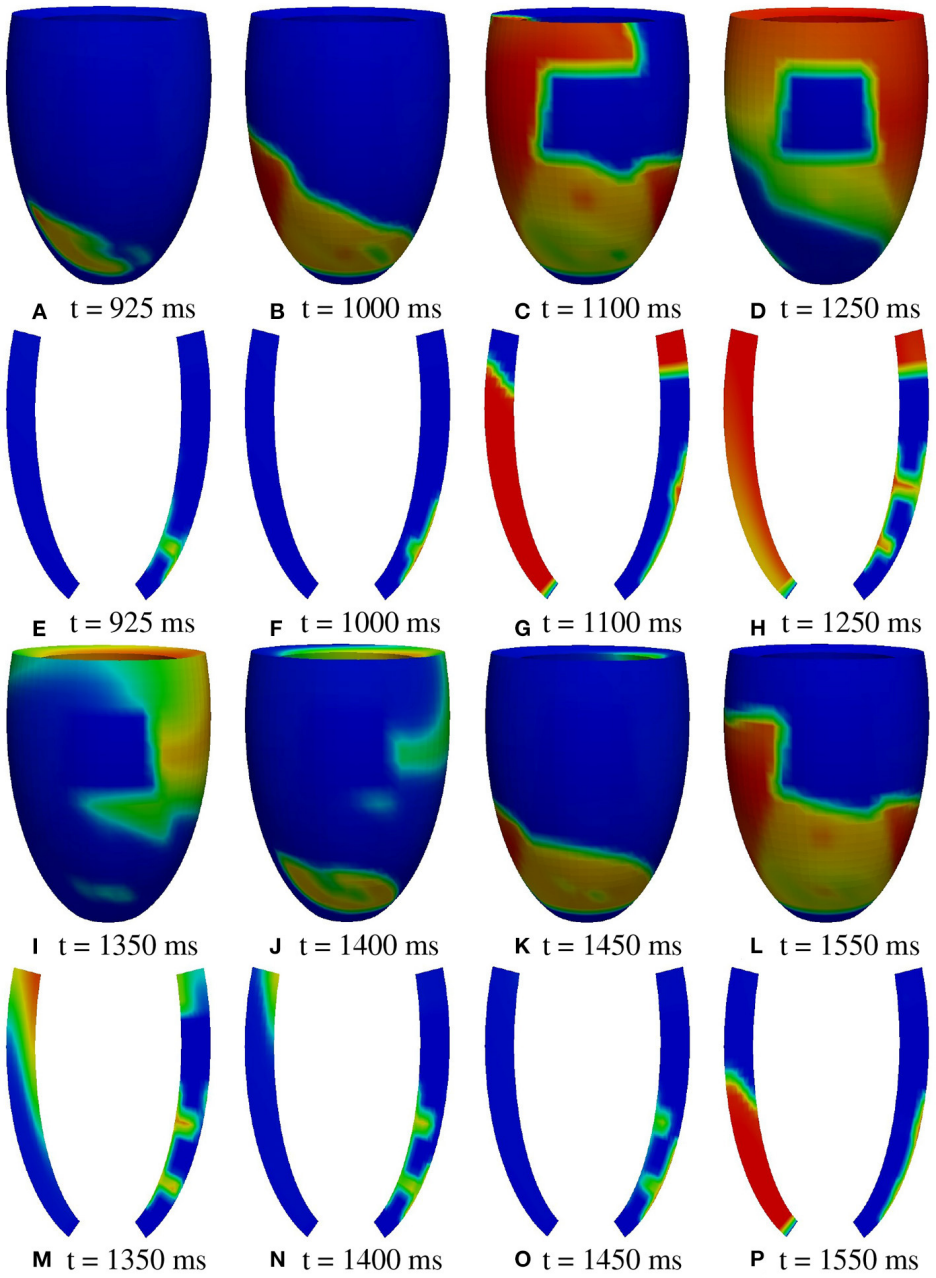
Patient #1. A2 stimulation. **(A–P)** Transmembrane potential snapshots (t = 925–1550 ms) on the epicardial surface and on a transmural section. t = 0 corresponds to the S1 stimulus. The S2 stimulus is applied at t = 350 ms. The colorbar is the same as in Figure 5.

**Figure 9:**
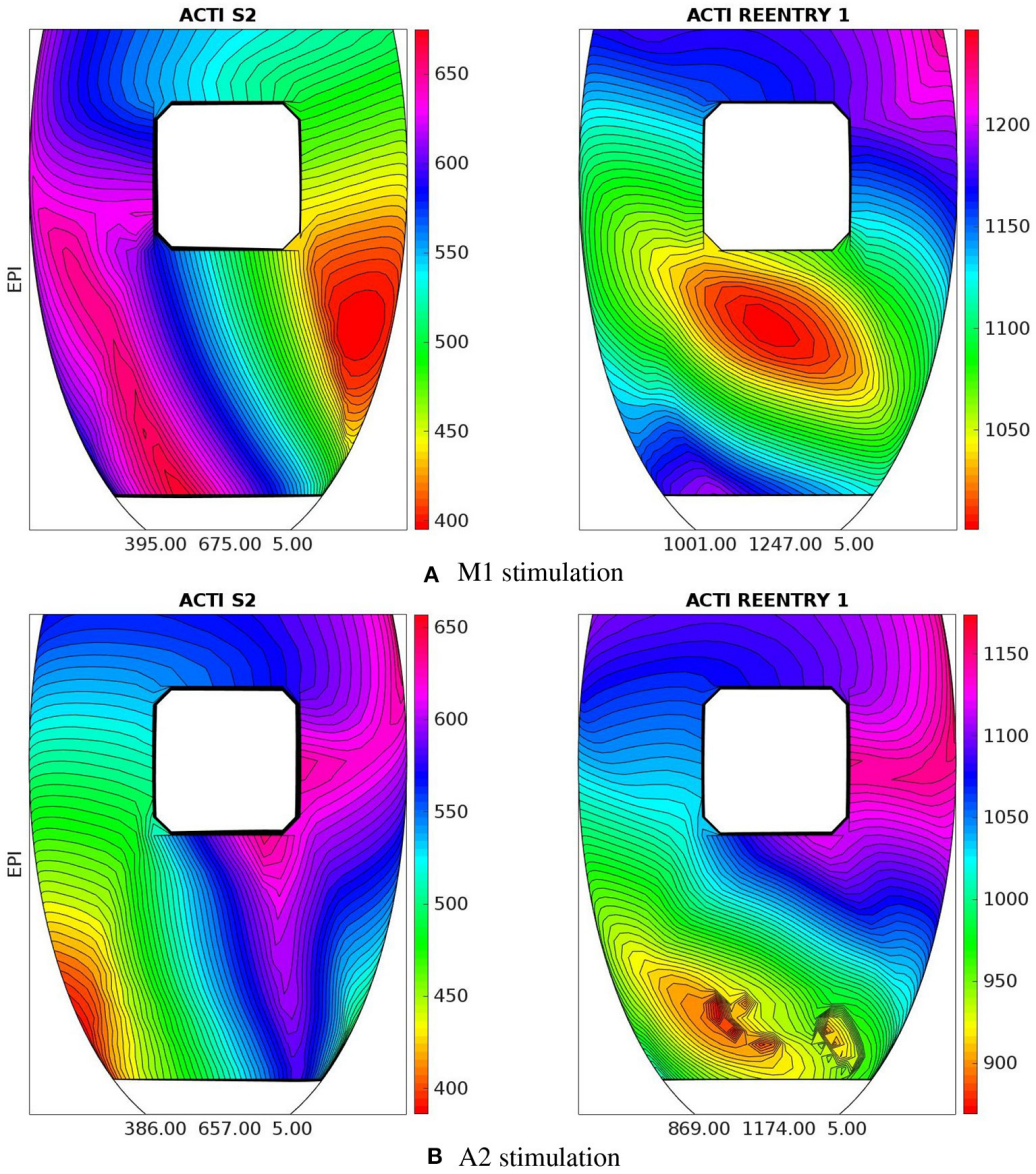
Patient #1. Epicardial activation time distributions of S2 and first reentrant excitation sequences for M1 **(A)** and A2 **(B)** stimulations. Below each panel is reported the minimum, maximum, and isochrones step in ms of the displayed map.

**A2 stimulation site**. Figures 7, 8 report some snapshots of the transmembrane potential on the epicardium and on two longitudinal transmural sections, after the S2 stimulus, applied at t = 350 ms, being t = 0 ms the onset of the S1 stimulus. At t = 450 ms (Figure 7A), the excitation layer elicited by the S2 stimulus spreads through the epicardial surface, entering the apical portion of BZ. Then, it proceeds toward the base, moving around the basal portion of the scar (Figures 7B–D). At t = 650 ms (Figure 7H), the endocardial excitation layer enters the apical isthmus and, propagates slowly through the isthmus from endocardium to epicardium (Figure 7O), it reaches the apical epicardial surface at t = 850 ms (Figure 7N). After the apical epicardial BKT (Figure 7J), excitation spreads through the epicardial BZ, generating the first cycle of re-entry (Figures 8A–H). The re-entrant activation then maintains, following the same circuit through the apical isthmus (Figures 8I–P) and thus inducing even in this case a sustained VT. We report in Figure 9B the epicardial activation time distributions of the S2 and first reentrant excitation sequence. In order to clarify the dynamics of the excitation sequence at the level of the epicardial surface and transmurally, we have added the movies **SM_paz1_A2_epi** and **SM_paz1_A2_trans** in the Supplementary Material.

A deeper investigation has shown that the apical isthmus is not able to directly generate a propagating excitation layer in the overlying epicardial area, but it releases an electrotonic current which flows into the subepicardial BZ layer, triggering the first re-entrant excitation localized on the left of the BZ (Figure 7N). The epicardial area overlying the apical isthmus is then excited by the re-entrant excitation at about t = 950 ms (Figure 8A). The same excitation phenomenon occurs during the second cycle of re-entry. In the subsequent cycles, instead, the current flowing through the isthmus is able to trigger directly the excitation when it reaches the epicardium.

**Figure 10:**
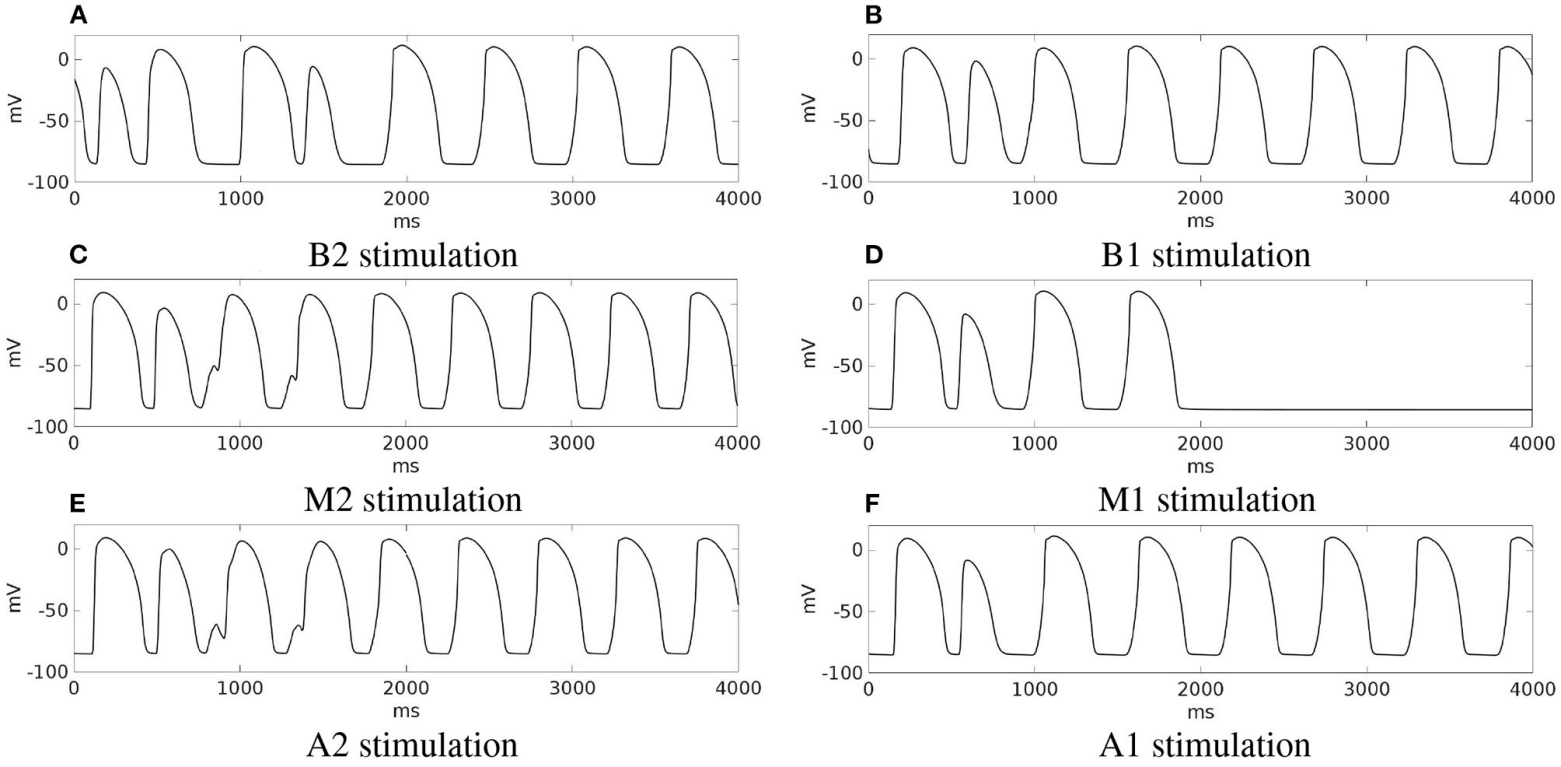
Patient #1. **(A–F)** Transmembrane potential waveforms in the six stimulation cases, computed from an epicardial size located in the BZ, at the exit of the transmural isthmus.

**Summary**. Stimulations from all the six pacing sites produced re-entry, which is sustained in all cases except from site M1. These outcomes are summarized in Figure 10, which reports for each simulation the transmembrane potential waveform in a sample epicardial point located in the BZ, at the exit of the transmural isthmus. We observed differences in the dynamics of re-entry among the stimulations. In stimulations B1, M1, A1, re-entry is triggered by an excitation layer that propagates through the central isthmus toward the epicardial surface. In stimulations B2, M2, A2, the pathway of re-entry follows the apical isthmus, but during the first two cycles of re-entry, the excitation propagating through the isthmus does not reach directly epicardial surface. Indeed, the electrotonic current flowing in the subepicardial BZ from the isthmus is able to trigger excitation. All subsequent cycles of re-entry are of the same type observed in stimulations B1, M1, and A1.

### 3.2. Patient #2 LV Model

**Figure 11:**
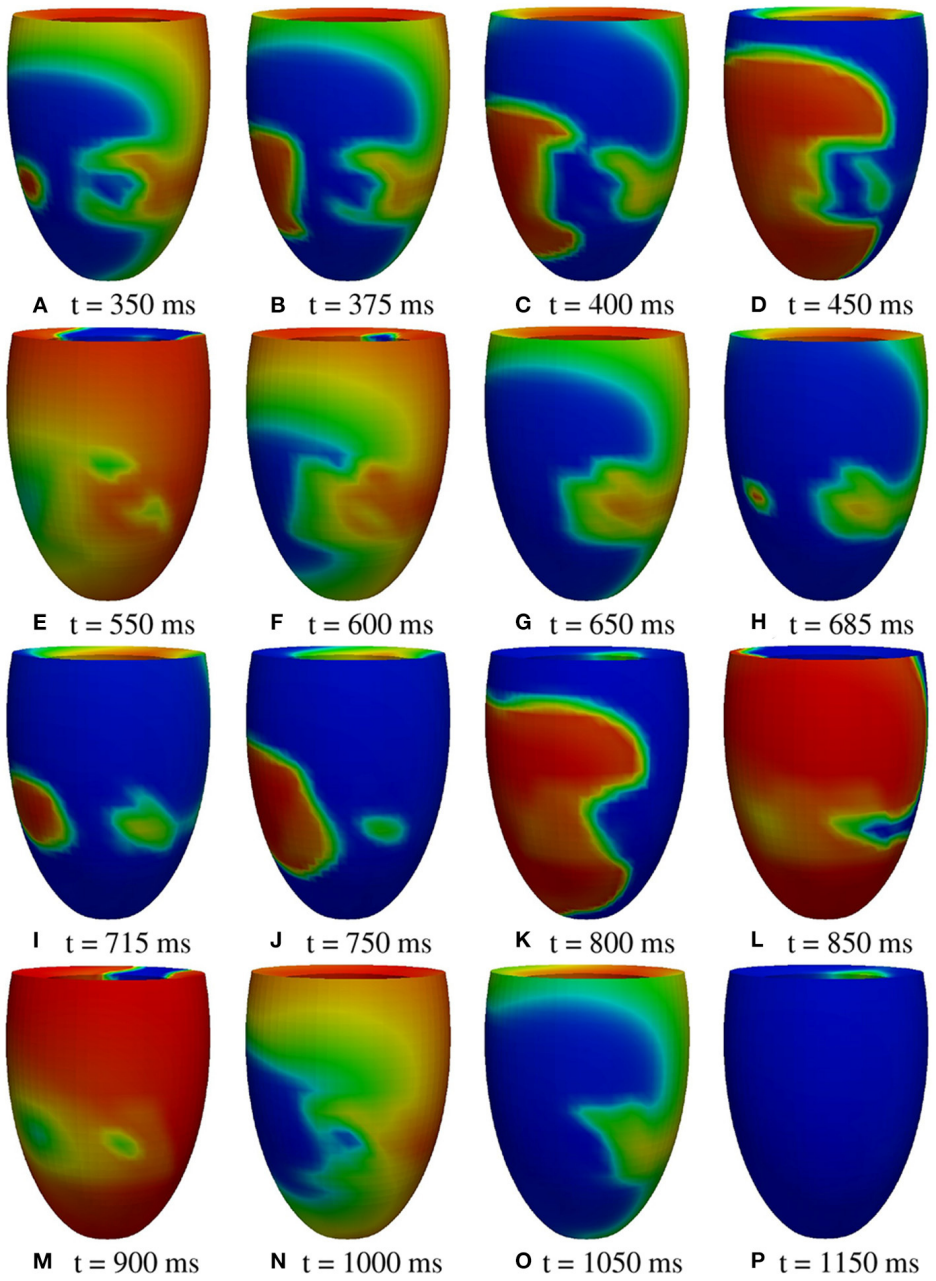
Patient #2. M2 stimulation. **(A–P)** Transmembrane potential snapshots (t = 350–1,150 ms) on the epicardial surface. t = 0 corresponds to the S3 stimulus. The S4 stimulus is applied at t = 300 ms. The colorbar is the same as in Figure 5.

**Figure 12:**
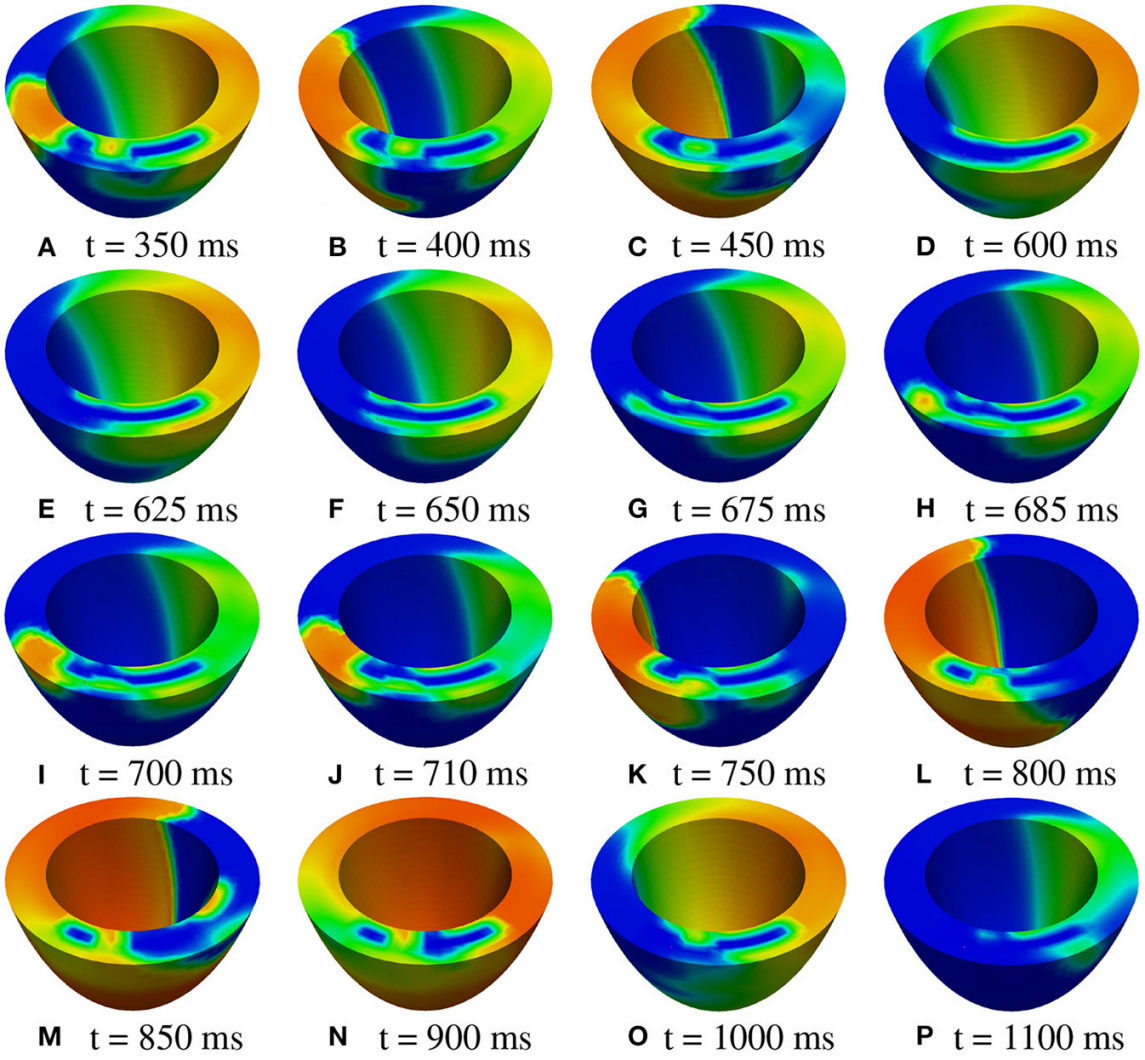
Patient #2. M2 stimulation. **(A–P)** Transmembrane potential snapshots (t = 350–1,100 ms) on a transmural section. t = 0 corresponds to the S3 stimulus. The S4 stimulus is applied at t = 300 ms. The colorbar is the same as in Figure 5.

**M2 stimulation site**. We apply an S2 stimulus with an S1-S2 coupling interval of 340 ms, an S3 stimulus with an S2-S3 coupling interval of 270 ms, and an S4 stimulus with an S3-S4 coupling interval of 300 ms. Figures 11, 12 report some snapshots of the transmembrane potential on the epicardium and on a circumferential transmural section, after the S4 stimulus, being t = 0 ms the onset of the S3 stimulus. A conduction block occurs when the excitation layer elicited by the S4 stimulation reaches the subepicardial BZ, at about t = 450 ms, as shown in Figure 12C. Then, the tissue in the subepicardial BZ layers in contact with the scar does not recover completely, and an excitation layer starts to propagate from right to left after t = 600 ms (Figures 12D–G). When this excitation layer reaches the left lateral border separating the BZ from the healthy tissue, it triggers an intramural re-entrant excitation propagating toward both the endocardium and epicardium (Figures 12H–J). Excitation then propagates through the whole LV tissue (Figures 11H–P), but re-entry is not sustained, since it dies after the first cycle.

**Summary**. An analogous non-sustained re-entry phenomenon occurs in the case of the M1 stimulation site. Instead, in case of A1 stimulation site, no re-entry is observed. From the other stimulation sites, we did not observe any sustained re-entry. Thus, irrespective of the stimulation site, sustained re-entry is never induced in the LV model of patient #2.

### 3.3. Modifications of Scar and BZ Configurations of Patients #1 and #2

In order to better understand the scar and BZ geometric features determining the different outcomes in the two patients, we considered the following three changes in the configuration of the BZ layer and/or the area of the isthmus section of patient#2:

- **configuration #1**: we reduce the section area of the isthmus, making it comparable with that of the central isthmus of patient #1;
- **configuration #2**: we make the subendo- and subepicardial BZ layer thickness as thin as in patient #1;
- **configuration #3**: we make the subendo- and subepicardial BZ layer thickness as thin as in patient #1 and we also reduce the section area of the isthmus, making it comparable with that of central isthmus of patient #1.

In **configuration #1**, after a sufficiently premature S2 stimulus applied at site M1, the epicardial excitation layer blocks at the exit of the isthmus, while the endocardial excitation layer enters the isthmus, propagating toward the epicardial surface. When it reaches the exit of the isthmus, it is unable to trigger excitation into the sub-epicardial BZ. For the next 30 ms, the myocardial volume is almost completely repolarized, but inside the isthmus the transmembrane potential maintains values above the threshold, generating an electrotonic current that flows in the sub-epicardial BZ layer and at 1,024 ms it is able to trigger a re-entrant excitation. However, excitation dies after the first cycle of re-entry (see the movie **SM_paz2mod_conf1_M1_epi** in the Supplementary Material).

In **configuration #2**, after the S2 stimulus, the excitation layer propagating through the isthmus is able to re-excite the subepicardial BZ layer, triggering a sustained re-entry of type A (see the movie **SM_paz2mod_conf2_M1_epi** in the Supplementary Material).

In **configuration #3**, after the S2 stimulus, the excitation layer propagating through the isthmus is able to re-excite the subepicardial BZ layer, triggering a sustained re-entry of type B (see the movie **SM_paz2mod_conf3_M1_epi** in the Supplementary Material).

**Figure 13:**
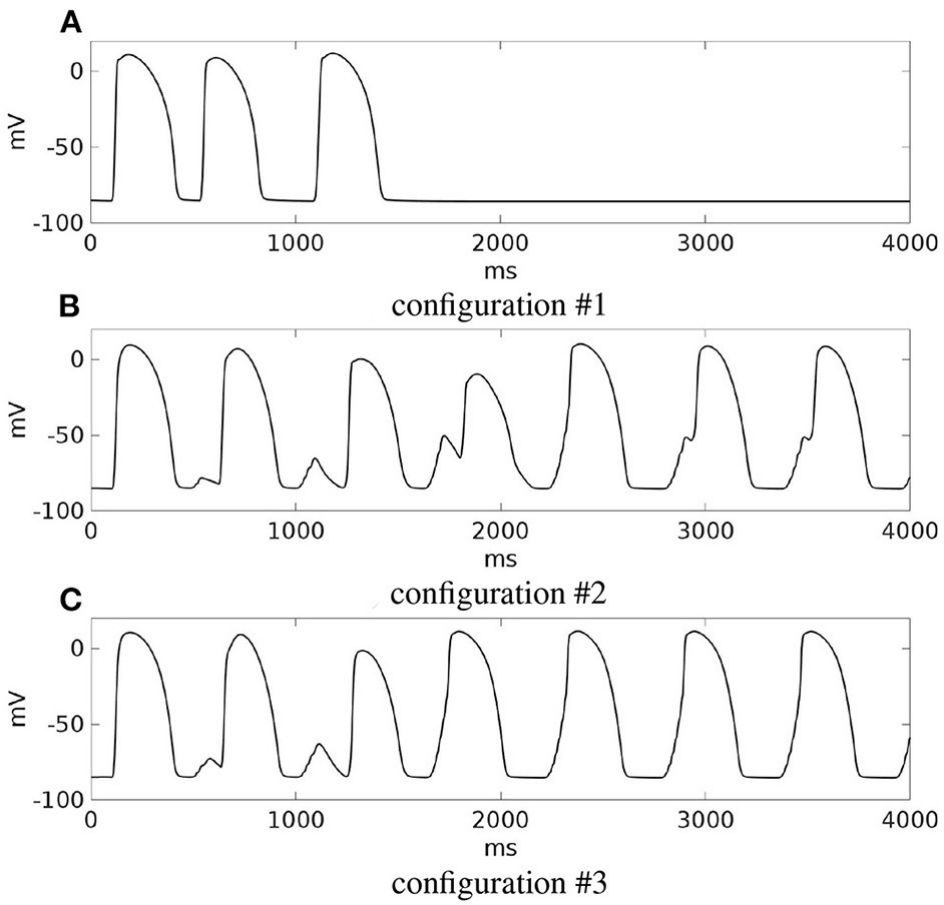
Modifications of patient #2. **(A–C)** Transmembrane potential waveforms in the three modified configurations, computed from an epicardial size located in the BZ, at the exit of the transmural isthmus. M1 stimulation.

The outcomes of the previous three configurations are summarized in Figure 13, which reports for each simulation, the transmembrane potential waveform in a sample epicardial point located in the BZ, at the exit of the transmural isthmus.

Finally, we modified the model of patient #1 by increasing the thickness of the subendo- and subepicardial BZ, making it comparable to that of patient #2. After a sufficiently premature S2 stimulus from site M2, we were able to induce a sustained re-entry (see the movie **SM_paz1mod_M2_epi** in the Supplementary Material).

## 4. Discussion

Improving arrhythmic risk stratification of patients with prior myocardial infarction and mildly reduced ejection fraction is one of the current challenges in clinical cardiology. Understanding the mechanisms (still unclear) and the scar geometric features that facilitate the onset of VT might help to identify patients at high risk.

In this work, two patients affected by infarct scar, both with LVEF > 35%, have been selected for EPS. Patient #1 resulted positive, whereas patient #2 was tested negative. On the basis of the information derived from their CMR data, we have developed a finite element model of the left ventricle, taking into account the position, extent, and topological features of the scars. Then, we have run numerical simulations based on the anisotropic Monodomain model of electrocardiology, mimicking an S1-S4 stimulation protocol, in order to

- verify whether the numerical modeling is able to reproduce the onset of VT in patient #1 and not in patient #2;
- ascertain the mechanisms that induce VT in patient #1;
- identify the geometric features of the infarct scar that facilitate the onset and maintenance of VT.

The simulation results have shown that, in patient #1, sustained VT occurs after a premature endocardial S2 stimulus. We were able to induce re-entry from all the six stimulation sites considered. Only in one case (M1 stimulation), the re-entry was not sustained. Depending on the location of the stimulus, the re-entrant circuit follows the path of the apical or central transmural isthmus. After the S2 stimulation, the excitation layer splits into two branches, one propagating through the subendocardial BZ and one through the subepicardial BZ. The mechanism that induces the onset of re-entry is the conduction block occurring when the epicardial excitation layer reaches the entrance of the apical or transmural isthmus, which is still refractory. The endocardial excitation layer instead, propagating slowly from the endocardial entrance through the isthmus, reaches the epicardial tissue, now excitable again, generating the re-entrant wave.

**Figure 14:**
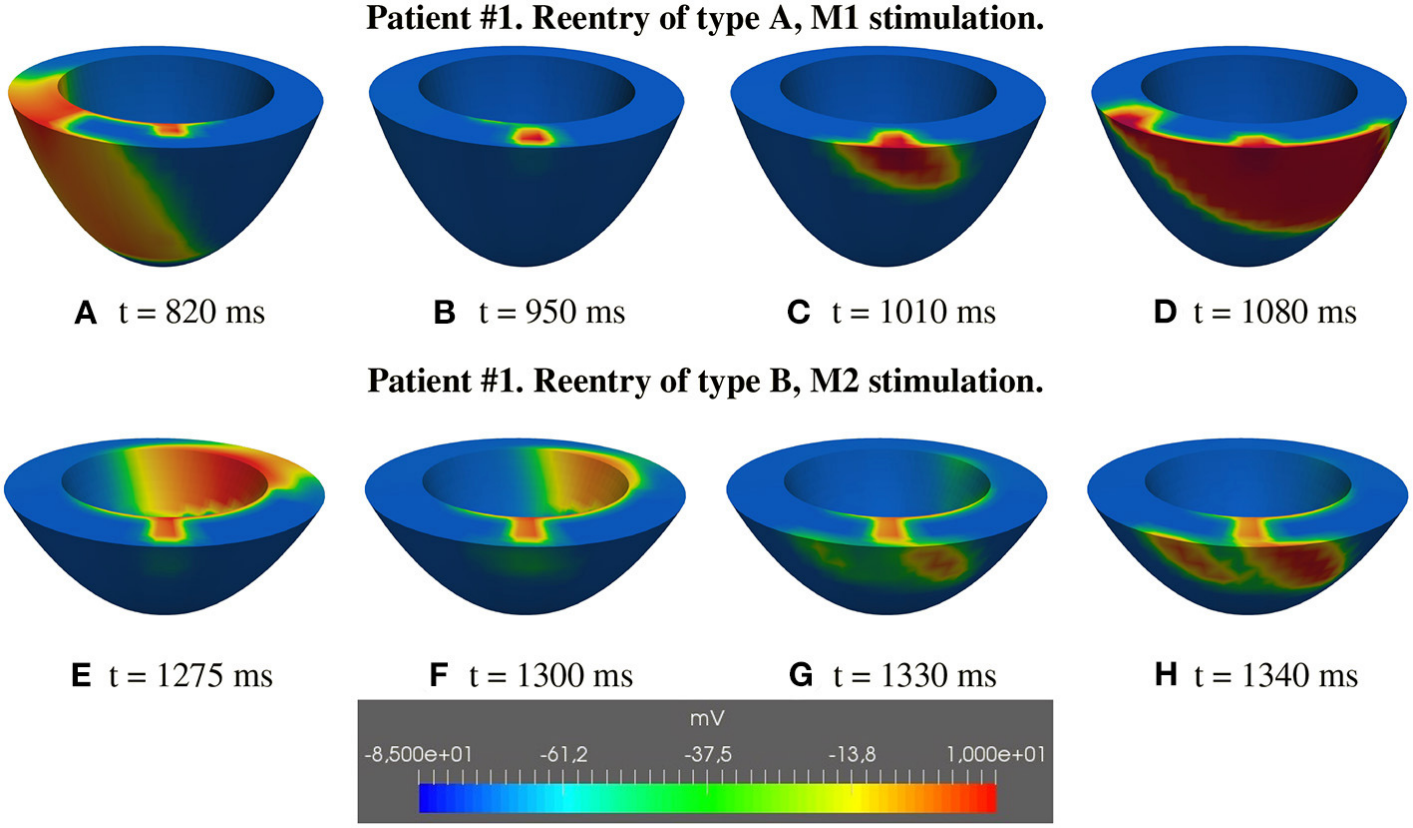
Patient #1. Reentry of types A and B. **(A–H)** Transmembrane potential snapshots on transmural sections across the central isthmus (First row) and across the apical isthmus (Second row). t = 0 corresponds to the S1 stimulus. The S2 stimulus is applied at t = 350 ms. **First row**: Reentry of type A related to M1 stimulation. **Second row**: Reentry of type B related to M2 stimulation.

We identified two different exits of the re-entrant pathway:

- type A: the excitation layer propagating in the isthmus reaches the epicardial tissue, now excitable again, generating the re-entrant wave propagating over the sub-epicardium and subsequently spreading in the whole tissue (Figures 5L, 6A,B, 14–first row);
- type B: the excitation layer propagating in the isthmus reaches the sub-epicardial BZ layer, but it is not able to elicit a propagating wavefront. The electrotonic load in the isthmus generates a current flowing through the sub-epicardial BZ layer mainly along the fiber direction.The accumulation of the electrotonic current near the two lateral borders between the BZ layer and the healthy tissue elicits two propagating excitation layers spreading over the sub-epicardium and in the whole tissue (Figures 7J–L, 14–second row).

Type A reentry is induced by stimulations B1, M1, A1, and the reentry circuit follows the central isthmus, whereas type B reentry is induced by stimulations B2, M2, A2, and the reentry circuit follows the apical isthmus. The different behavior can be attributed to:

- the slightly smaller section diameter of the apical isthmus with respect to the central one;
- the greater transmural thickness of the central wall with respect to the apical one;

In the LV model of patient #2, we did not observe the same mechanisms of reentry. In this case, only after the S4 stimulus, a conduction block occurs in the subepicardial BZ, generating a re-entrant wave that dies after the first cycle. Thus, we were not able to induce sustained VT in patient #2.

Consequently, the first conclusion of this study is that our computational results agree with the EPS performed on the two patients, confirming the effective predictive capability of numerical simulations in discriminating patients at high arrhythmic risk, as proposed in the pioneering papers (Ashikaga et al., 2013; Arevalo et al., 2016).

Analyzing the topology of the scar and BZ of the two patients, we note that in both cases, the scar is characterized by at least a transmural BZ isthmus, whereas the main differences between the two scar configurations are the following:

- the scar extent, since that of patient #1 occupies a portion of LV larger than that of patient #2;
- the isthmus thickness, since the isthmus of patient #2 is thicker than those of patient #1;
- the subendocardial and subepicardial BZs thickness, since those of patient #2 is significantly thicker than those of patient #1.

The scar extent plays an important role in determining VT, since patients with large scars are more likely to undergo VT than patients with small scars, as also observed in our previous work by Colli-Franzone et al. (2019). In order to understand whether also the thickness of the BZ plays a role in determining the onset and maintenance of sustained re-entry, we modified the configuration of the BZ layer and/or the area of the isthmus section of the two patients. The results have shown that, making the epicardial BZ of patient #2 as this as that of patient #1, even though maintaining the original small scar dimensions, sustained VT can be induced after the programmed stimulation protocol. On the other hand, in patient #1, increasing the thickness of the epicardial BZ does not alter significantly the inducibility of sustained VT.

Summarizing, the second conclusion of the present work is that scar configurations with transmural BZ isthmuses and subendo- and subepicardial BZ are likely to be arrhythmogenic. The presence of a transmural BZ isthmus is crucial to determine the onset and maintenance of re-entry since the pathway followed by the simulated re-entrant circuits always passes through the isthmus. However, its thickness does not seem to affect significantly the induction of re-entry. For sufficiently large scars, such as that of patient #1 (about 1400 *mm*^2^), sustained re-entry occurs irrespectively of the thickness of the subendo- and subepicardial BZs. On the other hand, for smaller scars, such as that of patient #2 (about 800 *mm*^2^), thin (less than 2 *mm*) subendo- and subepicardial BZs facilitate the onset and maintenance of re-entry. This result is in agreement with previous experimental studies reported in Peters et al. (1997) and Wit et al. (1982).

### 4.1. Clinical Implications

We identified at CMR a geometric pattern of scar and BZ, characterized by thin subendo- and subepicardial BZ and transmural BZ isthmuses, provides a major risk for sustained VT inducibility at EPS. This information is easy to use in daily routine and could be added to known non-invasive risk factors to identify patients with ischemic cardiomyopathy and moderate systolic disfunction to submit to EPS.

### 4.2. Limitations

In order to reduce the computational effort, the numerical simulations were performed considering the monodomain instead of the bidomain representation of the cardiac tissue and we neglected the presence of the Purkinje network, that might play a role in influencing the patterns of reentry.

We also considered only scar formations with compact fibrosis, treated as non-conductive obstacles. A recent study (Nezlobinsky et al., 2021) has focused on the influence of various non-compact fibrotic textiles on the arrhythmogenic substrate. The BZ in our study was modeled as an electrical homogeneous tissue, with uniform thickness, instead of a highly heterogeneous region, both from the electrical and geometric point of view; (Peters et al., 1997; Ciaccio et al., 2007).

Furthermore, the electro-mechanical coupling was disregarded in our study. For the inclusion of mechanical and hemodynamical models in the numerical simulations of arrhythmias, we refer to the recent paper (Salvador et al., 2021).

## Data Availability Statement

The raw data supporting the conclusions of this article will be made available by the authors, without undue reservation.

## Author Contributions

VG, RD, and CS have collected and analyzed the clinical data, planned, and written the manuscript. SS, PC, and LP have developed the mathematical model and numerical solver, performed the numerical simulations, planned, and written the manuscript. All authors contributed to the article and approved the submitted version.

## Funding

SS was supported by grants of MIUR (PRIN 2017AXL54F_003) and INdAM-GNCS. PC was supported by grants of INdAM-GNCS. LP was supported by grants of MIUR (PRIN 2017AXL54F_002) and INdAM–GNCS.

## Conflict of Interest

The authors declare that the research was conducted in the absence of any commercial or financial relationships that could be construed as a potential conflict of interest.

## Publisher's Note

All claims expressed in this article are solely those of the authors and do not necessarily represent those of their affiliated organizations, or those of the publisher, the editors and the reviewers. Any product that may be evaluated in this article, or claim that may be made by its manufacturer, is not guaranteed or endorsed by the publisher.

## Supplementary Material

The Supplementary Material consists of the text file **supplementary.docx** and eight movies:

SM_paz1_M1_epi Patient #1, M1 stimulation, evolution of the epicardial transmembrane potential distribution after the S1 stimulus (t = 0 ms) and the S2 stimulus (t = 350 ms). The reentry follows the central transmural isthmus and dies after two cycles.

SM_paz1_M1_trans Patient #1, M1 stimulation, evolution of the transmural transmembrane potential distribution after the S1 stimulus (t = 0 ms) and the S2 stimulus (t = 350 ms). The reentry follows the central transmural isthmus and dies after two cycles.

SM_paz1_A2_epi Patient #1, A2 stimulation, evolution of the epicardial transmembrane potential distribution after the S1 stimulus (t = 0 ms) and the S2 stimulus (t = 350 ms). The reentry follows the apical transmural isthmus and is sustained.

SM_paz1_A2_trans Patient #1, A2 stimulation, evolution of the transmural transmembrane potential distribution after the S1 stimulus (t = 0 ms) and the S2 stimulus (t = 350 ms). The reentry follows the apical transmural isthmus and is sustained.

SM_paz2mod_conf1_M1_epi Modification of patient #2, configuration #1, M1 stimulation, evolution of the epicardial transmembrane potential distribution after the S1 stimulus (t = 0 ms) and the S2 stimulus (t = 350 ms). The reentry dies after the first cycle.

SM_paz2mod_conf2_M1_epi Modification of patient #2, configuration #2, M1 stimulation, evolution of the epicardial transmembrane potential distribution after the S1 stimulus (t = 0 ms) and the S2 stimulus (t = 520 ms). The reentry follows the transmural isthmus and is sustained.

SM_paz2mod_conf3_M1_epi Modification of patient #2, configuration #3, M1 stimulation, evolution of the epicardial transmembrane potential distribution after the S1 stimulus (t = 0 ms) and the S2 stimulus (t = 520 ms). The reentry follows the transmural isthmus and is sustained.

SM_paz1mod_M2_epi Modification of patient #1, M2 stimulation, evolution of the epicardial transmembrane potential distribution after the S1 stimulus (t = 0 ms) and the S2 stimulus (t = 350 ms). The reentry follows the transmural isthmus and is sustained.
